# Special Issue “Synthetic Biology for Biosensing in Health and Environmental Applications”

**DOI:** 10.3390/bios13100937

**Published:** 2023-10-19

**Authors:** Baojun Wang, Cheemeng Tan

**Affiliations:** 1 College of Chemical and Biological Engineering & ZJU-Hangzhou Global Scientific and Technological Innovation Centre, Zhejiang University, Hangzhou 310058, China; 2 Department of Biomedical Engineering, University of California Davis, Davis, CA 95616, USA

Biosensors are analytical devices that utilize biological sensing elements, such as enzymes, antibodies, nucleic acids, or cells, to detect a given analyte. In general, biosensors could be divided into two subtypes. The first subtype involves the direct interaction or reaction between molecules or biomolecules. The second subtype relies on cellular and cell-free expression systems involving biological transcription–translation processes. Due to the advantages of easy construction, high specificity, and sensitivity, biosensors have been used for a wide range of applications such as portable disease diagnosis, environmental monitoring, and food safety. This Special Issue aims to cover the recent discovery, engineering, and translation of biosensors for biomedical and environmental applications.

In particular, synthetic biology has ushered in engineering approaches to design cells and cell-free expression systems for biosensing applications. To establish these applications, synthetic biological systems are subject to design, test, and build cycles to modify their gene and protein circuits for specific application objectives. Synthetic biology offers new tools and strategies to accelerate the development, improve the performance, and address the present limitations of cell-based and cell-free biosensors, which will facilitate their adoption as analytical devices in various settings.

Surface biosensors offer advantages such as high sensitivity, real-time performance, and the ability to detect a wide range of analytes. In this Special Issue, Nisar et al. developed a sensing platform to detect Cardiac troponin I, which is a prognosis factor for heart attack [1]. The sensing platform used anisotropic gold nanoclusters (AuNCs) with the anti-cTnI antibody (acTnI). The paper validated the immunosensor in vitro by adding different concentrations of cTnI to the artificial serum. The paper showed a wide detection range from 0.06 to 100 ng/mL. The results suggest that the platform could be used to screen cTnI in blood serum samples. In another paper in this Special Issue, Qiao et al. tackled the problem of detecting phosphoproteins in sweat for the early detection of various neurological diseases [2]. The paper examined a sensing platform for detecting phosphoprotein in sweat using an intercalation structure MXene@anatase/rutile TiO_2_ ternary heterojunction. In addition, the paper investigated the diffusion of phosphoprotein molecules in the ternary heterojunction structure using molecular dynamics simulation. They also verified the active sites in the MXene@anatase/rutile TiO_2_ ternary heterojunction and the synergistic effect of the heterojunction. This newly developed sensor showed promising results for detecting phosphoprotein in actual sweat samples.

Another type of biosensor leverages the biological machinery of whole cells. Miller et al. summarize the current strategies for improving the performance of small-molecule-responsive biosensors in bacterial cells [3]. They first review the different mechanisms for designing bacterial cell-based biosensors. Next, they present the various approaches that enhance sensor functionality, including ways of engineering the underlying biosensor genes, choosing the right genetic reporters, and tuning gene expression. They also discuss the incorporation of additional genetic modules, such as protein pumps for ligand export or accrual in cells, genetic circuits for amplifying input signals, and multi-input logic gating. In another research article in this Special Issue, Akboğa et al. studied the use of a recombinase-based genetic circuit in a heavy-metal-responsive *E. coli*-based biosensor system [4]. They harnessed the modularity of Bxb1 recombinase to improve the leakiness of the biosensor. In particular, they combined an engineered semi-specific heat shock response promoter with a specific cadmium-responsive promoter via the recombinase-expression-induced inverting of the cadmium-responsive output promoter. The authors further investigated the sensor performance under various conditions, including different media, cell inoculum, temperature, and pH. Finally, they identified the optimal working conditions leading to the increased fold change and shorter response time of the biosensor.

Different from whole-cell biosensors, cell-free biosensors leverage cellular machinery without cell membranes. Wang et al. introduce the concept of cell-free transcription–translation biosensors, in which the biosensing genetic elements are embedded in an abiotic cell-free gene expression system instead of living cells [5]. The resulting sensors will have: (1) high biosafety without the auto-replication of living cells, (2) fast material transport, (3) high sensitivity without the membrane barrier, and (4) long-term stability. They present the current strategies and latest progress towards improving the performance of cell-free biosensors and discuss the existing challenges, prospects, and efforts required to maximize the potential of such new sensing systems.

Biosensors for biomedical applications have made remarkable progress, driven by the demand for accurate and real-time diagnostic tools. Miniaturization has led to portable biosensors for point-of-care diagnostics [6], while multiplexing enables the simultaneous detection of multiple analytes [7]. Sensitivity and selectivity are enhanced through advancements in sensor design and signal amplification techniques [8]. The integration with microfluidics and artificial intelligence may enhance biosensor performance [9], potentially allowing for better sample handling and intelligent data analysis. New cellular chassis could also broaden the application scope of biosensors [10]. However, multiple challenges remain. One major challenge is ensuring the compatibility of biosensors with biological systems to minimize adverse reactions or interference with the target analytes [11]. Another challenge is ensuring biosensors’ long-term stability and reliability to guarantee consistent performance over time [8]. Factors such as sensor degradation, fouling, and drift in signals can affect the accuracy and precision of their measurements. Overcoming these challenges through interdisciplinary collaboration in synthetic biology is crucial for realizing biosensors’ potential in transforming biomedical diagnostics.
